# Supplementary material for: Comparative multiomics analysis of cell physiological state after culture in a basket bioreactor
Source: Sci Rep. 2022 Nov 23;12:20161. doi: 10.1038/s41598-022-24687-4 (PMC9686226; doi:10.1038/s41598-022-24687-4)
Supplement: Supplementary file 1 — Supplementary Information 1. [file 41598_2022_24687_MOESM1_ESM.zip › raw data/Transcriptomic raw data/6.Enrichment/1.GO/TE1vsTC1/ALL/TE1vsTC1.all_GObar_mf.pdf]

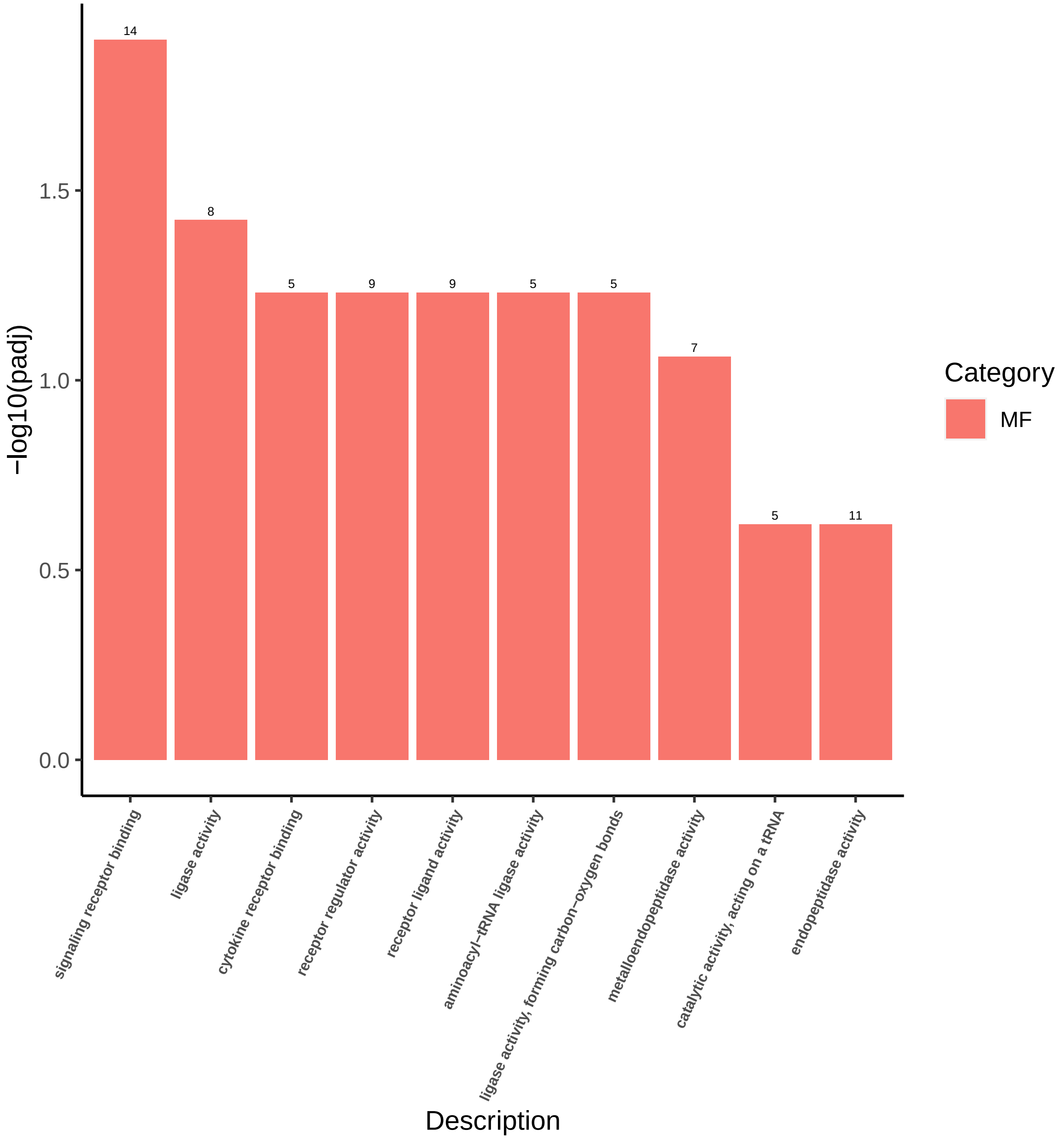

−log10(padj)
0.0
0.5
1.0
1.5
14
8
5
9
9
5
5
7
5
11
signaling receptor binding
ligase activity
cytokine receptor binding
receptor regulator activity
receptor ligand activity
aminoacyl−tRNA ligase activity
ligase activity, forming carbon−oxygen bonds
metalloendopeptidase activity
catalytic activity, acting on a tRNA
endopeptidase activity
Description
Category
MF
